# Significantly improved electrocatalytic oxygen reduction by an asymmetrical Pacman dinuclear cobalt(ii) porphyrin–porphyrin dyad†
†Electronic supplementary information (ESI) available: Fig. S1–S27, Table S1, and crystallographic data in CIF format for complexes **2** and **5**. The X-ray crystallographic coordinates for reported structures in this article have been deposited at the Cambridge Crystallographic Data Centre (CCDC), under deposition number CCDC 1956352 and 1956353. For ESI and crystallographic data in CIF or other electronic format see DOI: 10.1039/c9sc05041h


**DOI:** 10.1039/c9sc05041h

**Published:** 2019-11-04

**Authors:** Yanju Liu, Guojun Zhou, Zongyao Zhang, Haitao Lei, Zhen Yao, Jianfeng Li, Jun Lin, Rui Cao

**Affiliations:** a Key Laboratory of Applied Surface and Colloid Chemistry , Ministry of Education , School of Chemistry and Chemical Engineering , Shaanxi Normal University , Xi'an 710119 , China . Email: ruicao@ruc.edu.cn; b Department of Chemistry , Renmin University of China , Beijing 100872 , China; c College of Materials Science and Optoelectronic Technology , University of Chinese Academy of Science , Beijing 101408 , China

## Abstract

Pacman dinuclear Co^II^ triphenylporphyrin-tri(pentafluorophenyl)porphyrin **1** and dinuclear Co^II^ bis-tri(pentafluorophenyl)porphyrin **2**, anchored at the two *meso*-positions of a benzene linker, are synthesized and examined as electrocatalysts for the oxygen reduction reaction (ORR). Both dinuclear Co bisporphyrins are more efficient and selective than corresponding mononuclear Co^II^ tetra(pentafluorophenyl)porphyrin **3** and Co^II^ tetraphenylporphyrin **4** for the four-electron electrocatalytic reduction of O_2_ to water. Significantly, although the ORR selectivities of the two dinuclear Co bisporphyrins are similar to each other, **1** outperforms **2**, in terms of larger catalytic ORR currents and lower overpotentials. Electrochemical studies showed different redox behaviors of the two Co sites of **1**: the Co^III^/Co^II^ reduction of the Co-TPP (TPP = triphenylporphyrin) site is well-behind that of the Co-TPFP (TPFP = tri(pentafluorophenyl)porphyrin) site by 440 mV. This difference indicated their different roles in the ORR: Co^II^-TPFP is likely the O_2_ binding and reduction site, while Co^III^-TPP, which is generated by the oxidation of Co^II^-TPP on electrodes, may function as a Lewis acid to assist the O_2_ binding and activation. The positively charged Co^III^-TPP will have through-space charge interactions with the negatively charged O_2_-adduct unit, which will reduce the activation energy barrier for the ORR. This effect of Co-TPP closely resembles that of the Cu_B_ site of metalloenzyme cytochrome *c* oxidase (C*c*O), which catalyzes the biological reduction of O_2_. This work represents a rare example of asymmetrical dinuclear metal catalysts, which can catalyze the 4e reduction of O_2_ with high selectivity and significantly improved activity.

## Abstract 2

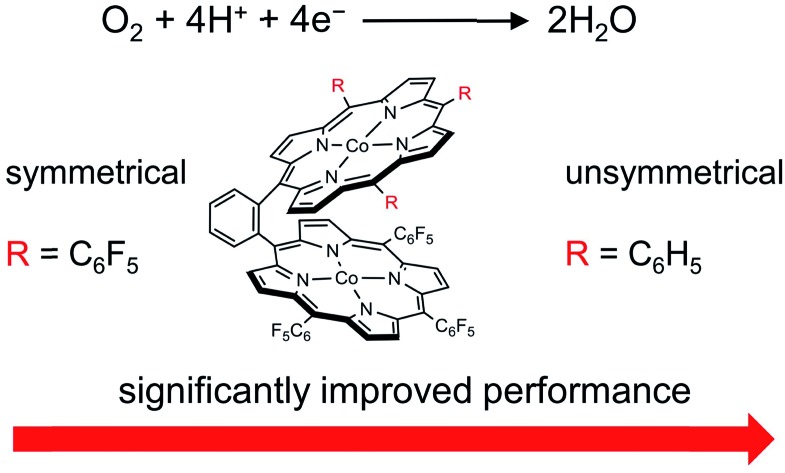
Asymmetrical Pacman dinuclear Co bisporphyrin shows significantly improved activity and selectivity for catalytic reduction of O_2_ to water in comparison with corresponding mononuclear Co porphyrins and symmetrical dinuclear Co bisporphyrins.

## Introduction

**Scheme 1:**
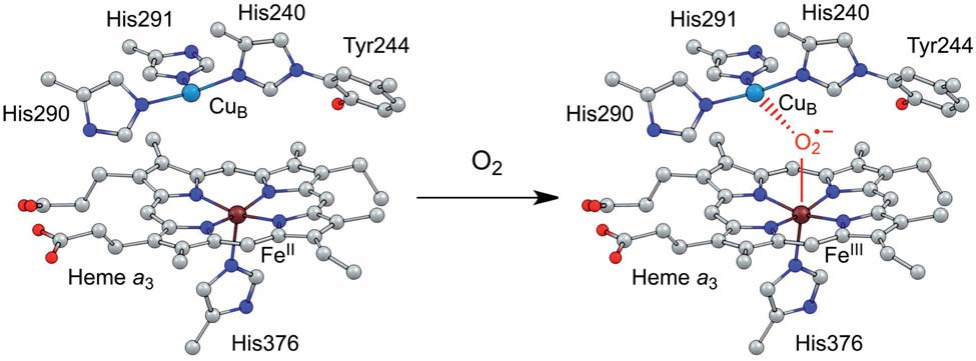
Illustration of the O_2_ binding and activation at the dinuclear heme *a*_3_ and Cu_B_ site of C*c*O.

Efficient and selective catalysts for the oxygen reduction reaction (ORR) are required in many kinds of energy conversion and storage devices, such as fuel cells and metal–air batteries, because the kinetically slow ORR is the key cathode reaction in these systems.^1^–^7^ Research on molecular catalysts is significant to provide fundamental knowledge on reaction mechanisms and structure–function relationships,^8^–^11^ which is essential for the development of new catalysts with improved performance. Among these studies, synthetic modeling of cytochrome *c* oxidase (C*c*O) has attracted particular attention.^12^–^16^ C*c*O belongs to a superfamily of heme/Cu oxidases and catalyzes the selective biological reduction of O_2_ to water at the dinuclear heme *a*_3_ and Cu_B_ site, which is the destination of electrons from the electron transport chain in respiration.^17^ The dinuclear heme/Cu site, in cooperation with the surrounding structures, including the tyrosine residue in the vicinity, the distal pocket, and also the *trans* ligand of the heme *a*_3_ Fe ion, is suggested to play crucial roles in minimizing the release of partially reduced oxygen species.^4^,^18^ Significantly, the Cu^I^ state of the Cu_B_ ion can provide an electron to reduce O_2_, and moreover, its Cu^II^ state can function as a Lewis acid to facilitate the O_2_ binding and activation at the heme site (Scheme 1).^16^

As inspired by nature, metal porphyrins have been extensively studied as ORR catalysts.^4^,^19^–^33^ In addition to biologically relevant Fe porphyrins, Co porphyrins have also received great attention because of their high activity and stability.^19^–^26^,^32^–^34^ However, mononuclear Co porphyrins are much less selective than Fe analogues to catalyze the 4e reduction of O_2_ to water. Unlike Fe complexes, Co complexes in general catalyze the 2e reduction of O_2_ because the peroxo intermediates of Co are challenging to undergo heterolytic O–O bond cleavage to generate terminal Co-oxo species that have large d electron counts and thus are high in energy.^35^–^37^ Several molecular design strategies have been reported to improve the selectivity for the 4e ORR, including (1) formation of intramolecular hydrogen bonding interactions to stabilize O_2_ adducts,^27^,^30^,^38^,^39^ (2) appending acid groups to assist proton delivery,^19^,^28^,^29^,^40^ and (3) introduction of functional groups to ensure rapid electron transfer between catalyst molecules and electrodes.^34^,^41^–^44^ Additionally, for complexes of late-transition metals, such as Co, it is found that dinuclear complexes are typically more efficient than mononuclear analogues to catalyze the 4e reduction of O_2_.^20^–^26^,^45^–^53^ This improvement is likely caused by the formation of peroxo-bridged dinuclear intermediates,^45^,^47^ whose subsequent reduction can result in the 4e ORR. Despite the improvement in the selectivity, however, from mononuclear to dinuclear complexes, the enhancement of catalytic activity, in terms of both catalytic currents and overpotentials, is unsatisfactory. In general, asymmetrical dinuclear complexes are rarely reported,^48^,^50^,^51^ while symmetrical dinuclear complexes are usually designed and studied with the two metal ions bearing very similar coordination environments. This will lead to parallel redox behaviors of the two metal ions. In other words, the two metal ions will be reduced at close potentials for O_2_ binding and activation. This will weaken the Lewis acid role of the second metal ion. Therefore, few dinuclear metal complexes can catalyze the ORR with both high activity and selectivity.

As mentioned above, in nature, the Lewis acidic and redox active features of the Cu_B_ site are essential for C*c*O to catalyze the ORR with both high activity and selectivity (Scheme 1). The presence of a positively charged Cu^II^ ion can decrease the activation energy barrier for the formation of the negatively charged O_2_-adduct unit at the heme site.^4^,^16^,^17^ Similar through-space charge interaction effects have been demonstrated for synthetic model complexes to catalyze artificial small molecule activation reactions,^54^,^55^ including O_2_ reduction and CO_2_ reduction. In order to develop ORR catalysts with satisfactory activity and selectivity, we herein report the synthesis and catalytic features of asymmetrical Pacman dinuclear Co^II^ triphenylporphyrin-tri(pentafluorophenyl)porphyrin **1**. Our results show that **1** is significantly more efficient and selective than corresponding mononuclear Co^II^ tetra(pentafluorophenyl)porphyrin **3** and Co^II^ tetraphenylporphyrin **4** to catalyze the 4e reduction of O_2_ to water, and more notably, it outperforms symmetrical dinuclear Co^II^ bis-tri(pentafluorophenyl)porphyrin **2**, in terms of both larger catalytic ORR currents and lower overpotentials. Electrochemical studies indicated that in **1**, Co^II^-TPFP is likely the O_2_ binding and reduction site, while Co^III^-TPP may function as a positively charged Lewis acid to assist the O_2_ binding and activation. This work represents a rare example of asymmetrical dinuclear metal catalysts with significantly improved performance for electrocatalytic 4e reduction of O_2_.

## Results

### Synthesis and characterization

**Fig. 1:**
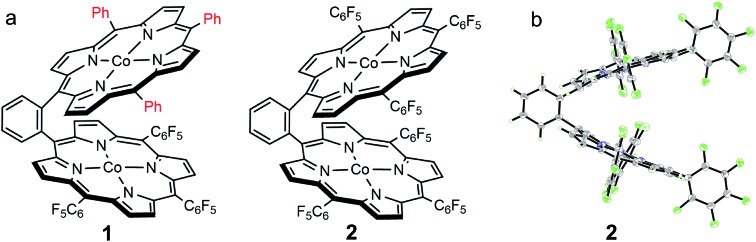
(a) Molecular structures of **1** and **2**. (b) Thermal ellipsoid plot of the X-ray structure of **2** (30% probability).

**Fig. 2:**
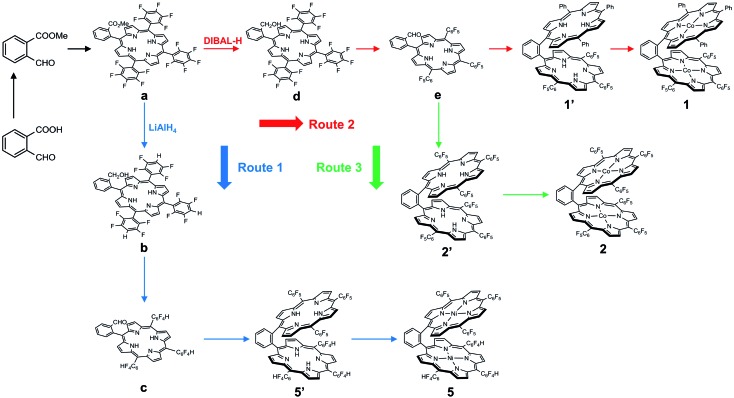
Synthetic routes to dinuclear metal complexes of **1**, **2**, and **5**.

We choose a benzene unit to link two porphyrin moieties at its two *meso*-positions (Fig. 1a). The resulting Pacman structure will enable the two porphyrin moieties to have a face-to-face configuration, which is considered to be suitable for the binding and activation of an O_2_ molecule.^20^,^21^ The synthetic routes to bisporphyrins and corresponding metal complexes are depicted in Fig. 2, and the details of synthetic conditions and characterizations are described in the ESI.† As shown in Fig. 2, methyl 2-formylbenzoate was used for the synthesis of intermediate **a** to introduce the first tri(pentafluorophenyl)porphyrin macrocycle (Fig. S2 and S3†). In order to reduce methyl benzoate to benzyl alcohol, we first treated **a** with lithium aluminium hydride (LiAlH_4_, route 1). However, in addition to the reduction of methyl benzoate, we found that the fluorine atom at the *para*-position of the three pentafluorophenyl units is also replaced by a hydrogen atom, leading to the formation of intermediate **b**. This undesired reduction was confirmed by ^1^H NMR spectroscopy and high-resolution mass spectrometry (HRMS). In ^1^H NMR, these three hydrogen atoms can be found with chemical shifts *δ* = 7.61 (Fig. S4†). The HRMS of **b** shows an ion with a mass-to-charge ratio of 861.1532 (Fig. S5†). This value matches the calculated value of 861.1518 for the monocation of [**b** + H^+^]^+^ with identical isotopic distribution. Subsequent oxidation of benzyl alcohol to benzaldehyde (Fig. S6 and S7†), and the next conversion to the second tri(pentafluorophenyl)porphyrin macrocycle resulted in bisporphyrin ligand **5′** (Fig. S8 and S9†). Attempts to obtain high quality single crystals of the dinuclear Co complex of **5′** were unsuccessful. However, we were able to get the single crystal X-ray structure of its dinuclear Ni analogue (see below), which clearly shows the replacement of three *para*-F atoms by H atoms.

In order to prevent this undesired replacement, we chose diisobutylaluminum hydride (DIBAL-H) to reduce intermediate **a**. With the use of this relatively weak hydride-donating reagent, we were able to selectively convert methyl benzoate to benzyl alcohol, giving intermediate **d**. Subsequent oxidation of benzyl alcohol to benzaldehyde leads to the formation of intermediate **e**. Both **d** and **e** are fully characterized by ^1^H NMR (Fig. S11 and S13,† respectively) and HRMS (Fig. S12 and S14,† respectively), confirming the preservation of the three pentafluorophenyl units during the treatment with DIBAL-H. Starting from **e**, we can synthesize the asymmetrical bisporphyrin ligand **1′** (Fig. S15,† route 2) and symmetrical analogue **2′** (Fig. S18,† route 3). The identity and purity of both **1′** and **2′** are confirmed by using ^1^H NMR spectroscopy and HRMS. In HRMS, **1′** displays an ion with a mass-to-charge ratio of 1421.3137 (Fig. S16†), matching the calculated value of 1421.3131 for the monocation of [**1′** + H^+^]^+^; **2′** displays an ion with a mass-to-charge ratio of 1691.1699 (Fig. S19†), matching the calculated value of 1691.1718 for the monocation of [**2′** + H^+^]^+^.

Reaction of **1′** with cobalt chloride CoCl_2_ gave dinuclear Co bisporphyrin **1** with a yield of 87%. The UV-vis spectrum of **1** in dimethylformamide shows a characteristic Soret band at 412 nm and a Q band at 560 nm (Fig. S21†). These bands are in accordance with those from corresponding mononuclear Co porphyrins **3** and **4**. In HRMS, **1** shows an ion with a mass-to-charge ratio of 1535.1461 (Fig. S17†). This value matches the calculated value of 1535.1437 for the monocation of [**1** + H^+^]^+^ with identical isotopic distribution. Reaction of **2′** with CoCl_2_ resulted in **2** with a yield of 85%. The UV-vis spectrum of **2** is similar to that of **1**, showing a Soret band at 401 nm and a Q band at 545 nm (Fig. S21†). The HRMS of **2** shows an ion with a mass-to-charge ratio of 1805.0038 (Fig. S20†), matching the calculated value of 1805.0023 for the monocation of [**2** + H^+^]^+^. All these results are in agreement with the expected structures for **1** and **2**. Notably, we were able to characterize the structure of **2** by using the single crystal X-ray diffraction method (see below), which confirmed the dinuclear Co bisporphyrin structure.

### Crystallographic studies

Although we were not able to structurally characterize complex **1**, its key structural information could be obtained from crystallographic studies of its analogues **2** and **5**. Single crystals of **2** that are suitable for X-ray structure analysis were obtained by slow vapor diffusion of hexane to a toluene solution of **2** at room temperature for two weeks. Complex **2** crystallized in the triclinic space group *P*1[combining macron] with *Z* = 2. Crystal data and structure refinement details can be found in Table S1.† In the X-ray structure of **2**, two symmetrical TPFP moieties are connected together at the two *meso*-positions of a benzene linker, providing a Pacman configuration (Fig. 1b). In each TPFP moiety, the three pentafluorophenyl substituents are well resolved. Particularly, the three *para*-F atoms can be well defined. This result further confirms the preservation of the three pentafluorophenyl units during synthesis. Each TPFP ligand coordinates a Co ion at the center of the porphyrin macrocycle through four N atoms. The Co–N bond distances are in the range of 1.908(2) Å to 2.007(2) Å. There are no axial ligands on the Co ions, leading to a distorted square-planar coordination geometry.

The bond valence sum (BVS) calculation suggested the d^7^ Co^II^ oxidation state. This result is consistent with the charge balance calculation because the porphyrin ligands are doubly negatively charged and no more counter anions are found in the X-ray structure of **2**. In addition, we measured the electron paramagnetic resonance (EPR) spectrum of **1** and **2** in toluene (Fig. S22† and 3a, respectively). The axial spectra confirmed the *S* = 1/2 low spin states of the four-coordinated Co^II^ porphyrin complexes with *g* = 2.26. The parallel components display a septet superhyperfine splitting pattern, which is due to the interaction of Co^II^ with N atoms. The Co···Co distance is 5.762 Å. Interestingly, in the Pacman cleft of **2**, there is a toluene solvent molecule, which is located at and is parallel to the angle bisector of the two porphyrin macrocycles.

**Fig. 3:**
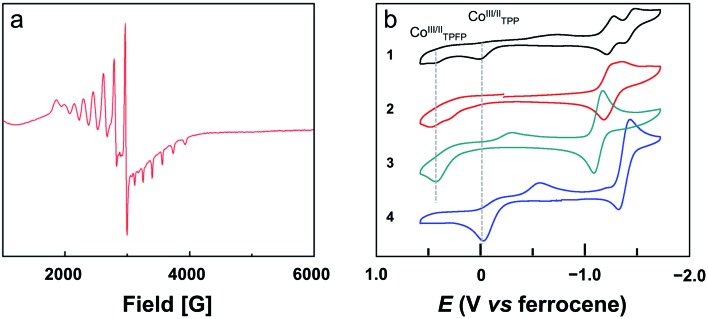
(a) EPR spectrum of **2** in toluene at 90 K. (b) CVs of 0.5 mM benzonitrile solutions of **1** and **2**, and 1.0 mM benzonitrile solutions of **3** and **4**. Conditions: GC working electrode, 0.1 M (Bu_4_N)PF_6_ benzonitrile solution, scan rate 50 mV s^–1^.

Moreover, we obtained high-quality single crystals of dinuclear Ni bisporphyrin **5** by slow vapor diffusion of pentane to a chloroform solution of **5** at room temperature for two weeks. Complex **5** crystallized in the triclinic space group *P*1[combining macron] with *Z* = 2 (see Table S1† for crystal data and structure refinement details). The structure of **5** closely resembles that of **2** (Fig. S23†), except that, in one TPFP moiety, the *para*-F atom of the three pentafluorophenyl substituents is each replaced by a *para*-H atom. This result is consistent with the findings in synthesis as mentioned above. Each Ni ion is located at the center of a porphyrin ligand with Ni–N bond distances ranging from 1.931(4) Å to 1.947(4) Å. The distorted square-planar coordination geometry of Ni and the BVS calculation all suggest the d^8^ Ni^II^ electronic configuration. The Ni···Ni distance is 6.182 Å. In the Pacman cleft of **5**, there is a pentane solvent molecule, which is located at the angle bisector of the two porphyrin macrocycles.

### Electrochemical and electrocatalytic ORR studies

We next measured the cyclic voltammograms (CVs) of dinuclear and mononuclear Co complexes in benzonitrile using a glassy carbon (GC) working electrode. The CV of mononuclear Co porphyrin **3** shows a Co^II^-to-Co^III^ oxidation wave at *E*_p,a_ = 0.43 V *versus* ferrocene (all potentials reported in benzonitrile in this work are referenced to ferrocene) and its corresponding reduction wave at *E*_p,c_ = –0.29 V (Fig. 3b). This irreversibility of the Co^III^/Co^II^ redox couple is indicative of the binding and dissociation of solvent benzonitrile on the Co ion during the Co^III^/Co^II^ conversion.^56^ Additionally, there is a reversible 1e reduction wave at *E*_1/2_ = –1.13 V, which is assigned to the Co^II^/Co^I^ redox couple. For mononuclear complex **4**, the Co^II^-to-Co^III^ oxidation wave is at –0.03 V, and its two corresponding reduction waves are at –0.15 and –0.56 V. The reversible Co^II^/Co^I^ redox couple is found at *E*_1/2_ = –1.38 V. As compared to **3**, the Co^II^-to-Co^III^ oxidation wave of **4** is shifted to the cathodic direction by 460 mV. This large cathodic shift is caused by the replacement of four strong electron-withdrawing pentafluorophenyl units by four phenyl units.^56^

Dinuclear Co complex **1** displays two Co^II^-to-Co^III^ oxidation waves at *E*_p,a_ = 0.43 and –0.03 V using the GC working electrode in benzonitrile. These values are identical to those represented by mononuclear complexes **3** and **4**, respectively. On the basis of this comparison, we can assign the oxidation wave at *E*_p,a_ = 0.43 V to the Co^II^-to-Co^III^ process of Co-TPFP, and assign the oxidation wave at *E*_p,a_ = –0.03 V to the Co^II^-to-Co^III^ process of Co-TPP. There are two reversible 1e reduction waves at *E*_1/2_ = –1.25 and –1.43 V, which can be assigned to the Co^II^/Co^I^ redox couple of Co-TPFP and Co-TPP, respectively. For dinuclear Co complex **2**, there are two close Co^II^-to-Co^III^ oxidation waves at *E*_p,a_ = 0.45 and 0.30 V. The two reversible 1e reduction waves of **2** are overlapped with *E*_1/2_ = –1.25 V. These results suggest that the electrochemical behaviors of the two Co porphyrin moieties in dinuclear complexes **1** and **2** are mainly determined by the electronic structures of the porphyrin macrocycles and that the interaction between the two porphyrin moieties is weak, which is consistent with the large Co···Co distance as revealed by crystallographic studies.

**Fig. 4:**
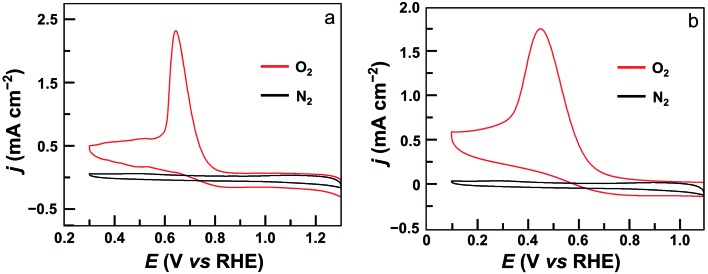
CVs of GC electrodes loaded with 5 μL ink of **1** (a) and **2** (b) under N_2_ (black) and O_2_ (red) in 0.5 M H_2_SO_4_ solution. Conditions: GC working electrode, 50 mV s^–1^ scan rate, 20 °C.

The electrocatalytic ORR features of complexes **1**–**4** loaded on carbon nanotubes were examined in 0.50 M H_2_SO_4_ aqueous solutions at room temperature. Their CVs under N_2_ and O_2_ clearly show that all four complexes are active for the ORR (Fig. 4 and S24†). Significantly, the CV of **1** displayed a large catalytic wave with the half-wave potential *E*_1/2_ = 0.72 V *versus* the reversible hydrogen electrode (RHE, all potentials reported in aqueous solutions in this work are referenced to RHE) (Fig. 4a). This value suggests that **1** represents the state-of-the-art electrocatalytic ORR performance among reported molecular catalysts operating under similar acidic conditions (see Table 1 for details). For complex **2**, the ORR half-wave potential *E*_1/2_ is 0.55 V (Fig. 4b), while for **3** and **4**, these values are *E*_1/2_ = 0.46 V and 0.41 V, respectively. Two conclusions can be made from these results. First, mononuclear complex **3** is superior to **4** for ORR catalysis, which is a result of the strong electron-withdrawing pentafluorophenyl substituents in **3**. Second, dinuclear Co catalysts are more efficient than mononuclear analogues, and **1** outperforms all other Co catalysts examined in this work. Considering that complex **2** contains more pentafluorophenyl substituents than **1**, the much higher efficiency of **1** is noteworthy.

**Table 1:** Comparison of catalytic performances of Co-porphyrin ORR electrocatalysts

Catalyst category	Catalyst	Solution	*η* at *E*_1/2_^*a*^ (V)	*n*	Ref.
Dinuclear Co bisporphyrin dyads	**1**	0.5 M H_2_SO_4_	0.51	3.9	**This work**
	**2**		0.70	3.8	
	Co_2_(DPX)	0.5 M HClO_4_ and 1.5 M CF_3_COOH	0.65	3.7	21 and 22
	Co_2_(DPD)		0.66	3.8	
	Co_2_(DPXM)	0.5 M HClO_4_ and 1.5 M CF_3_COOH	0.75	3.6	20
	Co_2_(DPDM)		0.74	3.5	
	Ox-Co	0.5 M H_2_SO_4_	0.84	3.5	25
	Oxa-Co		0.70	3.9	
	Benzo-Co		0.87	3.2	
	Co_2_PP_0_	pH 6.8 phosphate buffer	0.93	2.3	57
	Co_2_PP_2_		0.94	2.2	
	Co_2_PP_5_		0.77	2.0	
	Co_2_PP_8_		0.81	1.9	
Dinuclear Co biscorrole dyads	(BCA)Co_2_	1 M HClO_4_	0.60	3.4	48
	(BCB)Co_2_		0.61	2.4	
	(BCX)Co_2_		0.61	2.9	
	(BCO)Co_2_		0.64	3.4	
	(BCS)Co_2_		0.64	3.1	
Dinuclear Co porphyrin-corrole dyads	(PCA)Co_2_	1 M HClO_4_	0.52	3.9	48
	(PCB)Co_2_		0.53	3.7	
	(PCX)Co_2_		0.54	3.7	
	(PCO)Co_2_		0.58	3.5	
	(PCO_X_)Co_2_		0.59	3.1	50
	(PMes_2_CX)Co_2_		0.67	2.5	
	(PMes_2_CO)Co_2_		0.67	2.4	
	(PMes_2_CO_X_)Co_2_		0.66	2.5	
Heterobimetallic Fe/Mn porphyrin-Co corrole dyads	(PCA)FeClCoCl	1 M HClO_4_	0.60	2.8	51
	(PCO)FeClCoCl		0.64	2.8	
	(PCX)FeClCoCl		0.66	2.6	
	(PCB)FeClCoCl		0.69	2.6	
	(PCX)MnClCoCl		0.66	2.6	
	(PCO)MnClCoCl		0.63	2.5	
	(PCB)MnClCoCl		0.67	2.8	
Mononuclear Co porphyrin	**3**	0.5 M H_2_SO_4_	0.73	2.9	**This work**
	**4**		0.83	2.9	

^*a*^Half-wave overpotential. Although the overpotentials for the 2e and 4e ORR are different, for simplicity, we treated all entries as the 4e ORR for overpotential calculation.

**Fig. 5:**
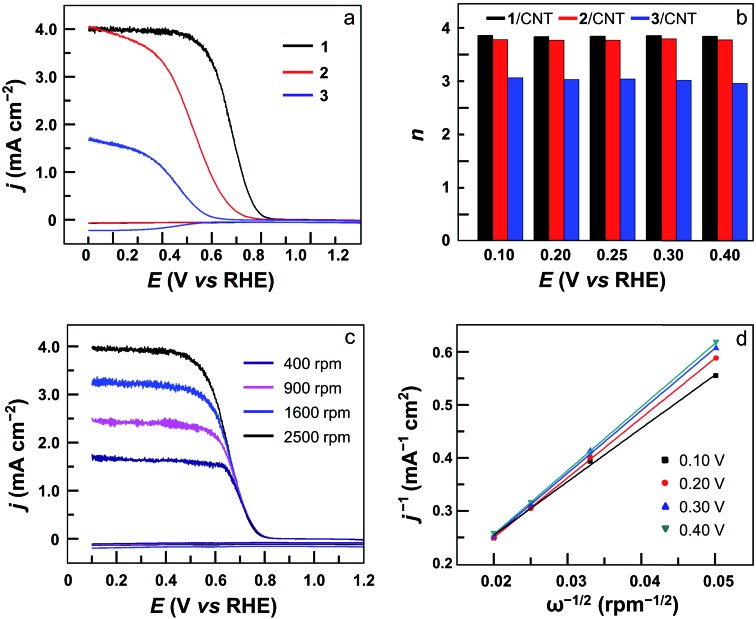
(a) RRDE measurements for the ORR at the GC disk electrode coated with **1** (black), **2** (red), and **3** (blue) in an O_2_-saturated 0.5 M H_2_SO_4_ solution at 2500 rpm. The ring electrode was polarized at 1.0 V. (b) *n* value of the ORR with **1** (black), **2** (red), and **3** (blue). (c) RRDE measurements for the ORR with **1** in O_2_-saturated 0.5 M H_2_SO_4_ solution at various rotation rates. (d) K–L plots for the ORR with **1**. Conditions: GC disk electrode (area 0.125 cm^2^), Pt ring electrode (area 0.188 cm^2^), 10 mV s^–1^ scan rate, 20 °C.

The ORR selectivity was then evaluated by determining the number of electrons *n* transferred per O_2_ molecule using rotating disk electrode (RDE) and rotating ring-disk electrode (RRDE) measurements. In the RRDE, the partially reduced oxygen species (*i.e.*, H_2_O_2_) that is possibly produced during the ORR at the GC disk electrode can be detected at the Pt ring electrode (Fig. 5a and S25†). According to the current densities detected at the disk electrode and the ring electrode, the *n* value can be determined to be 3.90 for **1**, 3.80 for **2**, 2.90 for **3**, and 2.90 for **4** (Fig. 5b). This result shows that in addition to much improved activity, dinuclear Co complexes **1** and **2** are also more selective than mononuclear analogues **3** and **4** to catalyze the 4e ORR. Moreover, the *n* value can be evaluated using the Koutecky–Levich (K–L) method in RDE measurements (Fig. 5c, S26 and S27†), giving *n* = 3.70 for **1**, 3.60 for **2**, 2.60 for **3**, and 2.60 for **4**. These values are in good agreement with those calculated from RRDE experiments. Under applied potentials of 0.10–0.40 V with catalyst **1**, the linear plots of *j*^–1^ (*j* = current density) on *ω*^–1/2^ (*ω* = the rotation rate) and the near-parallelism of these fitting lines suggested a first-order reaction dependence on dissolved O_2_ concentrations and similar ORR *n* values under different potentials (Fig. 5d).

The improved selectivity of dinuclear Co complexes for the 4e ORR shows that the Pacman structure constructed at the two *meso*-positions of a benzene linker enables efficient cooperation between the two Co ions. For late-transition metals, such as Co, the electrostatic repulsion between their d electrons and the p electrons of the oxo ligand will make terminal late-transition metal-oxo species unfavorable from an energy point of view. As a consequence, mononuclear Co complexes in general catalyze the 2e ORR, because the key peroxo intermediates are challenging to undergo heterolytic O–O bond cleavage to generate high-energy terminal Co-oxo species. Nevertheless, the bimetallic mechanism with the formation of peroxo-bridged dinuclear Co complexes is likely to happen. Subsequent homolytic O–O bond cleavage can lead to the improved 4e ORR.

## Discussion

Despite that dinuclear Co complexes **1** and **2** are both highly selective for the 4e reduction of O_2_, the superior activity of **1** is noteworthy. Strong electron-withdrawing groups, such as the *meso*-pentafluorophenyl substituents, can cause large shifts of the redox couples to the anodic direction and thus are usually favored for ORR catalysis,^58^,^59^ because this effect will lead to the formation of catalytically active species at relatively smaller overpotentials. Complex **2** has two Co-TPFP units, while complex **1** has one Co-TPFP unit and one Co-TPP unit. The Co^III^/Co^II^ reduction of the Co-TPP site of **1** is well behind that of the Co-TPFP site by 440 mV, which is in agreement with the replacement of three pentafluorophenyl groups by three phenyl groups. Although the two Co sites in **2** are more easily reduced to the catalytically active Co^II^ state, the half-wave overpotential of **2** is larger than that of **1** by 165 mV. Additionally, to reach the plateau currents, the potential required by **2** is larger than that of **1** by >300 mV. These results are in sharp contrast to the fact that **2** has more electron withdrawing groups.

**Fig. 6:**
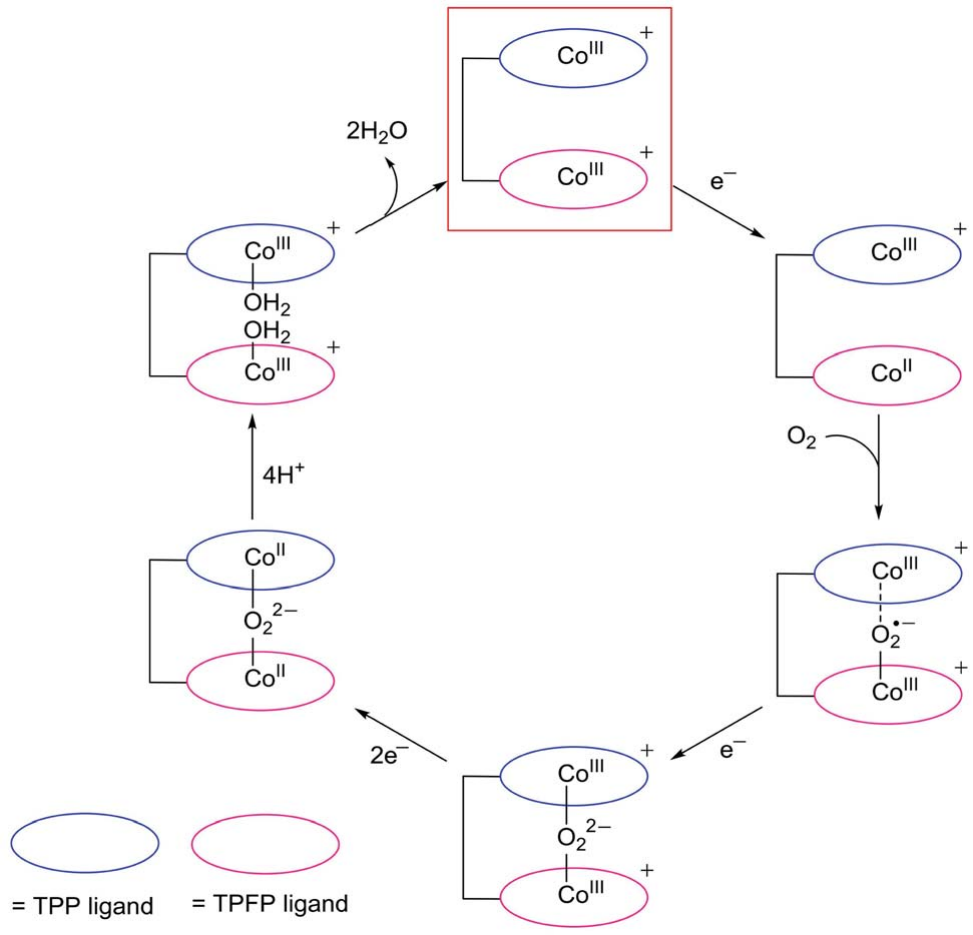
Proposed catalytic cycle for the ORR with **1**. Complex **1** will be instantly oxidized to the Co^III^Co^III^ oxidation state on the electrode upon applying large positive potentials, and this 2e-oxidized species (highlighted with a red box) is the real starting catalyst.

On the basis of these results, we propose that, upon reduction to the Co^II^ state, the Co-TPFP site of **1** becomes catalytically active for binding and activating the O_2_ molecule,^4^,^48^,^50^ while at this stage, the Co-TPP site of **1** still maintains the Co^III^ oxidation state (Fig. 6). The reaction of Co^II^-TPFP and O_2_ leads to the formation of a formally Co^III^–O_2_˙^–^ species.^60^,^61^ This negatively charged O_2_-adduct unit is likely to interact with the positively charged Co^III^-TPP unit. Such through-space charge interaction effects have been demonstrated to assist the formation and stabilization of charged intermediates.^54^,^55^ As a consequence, the Co^III^-TPP unit is suggested to be able to decrease the activation energy barrier for the binding and activation of O_2_ in the Pacman cleft of **1**. However, for complex **2**, its two Co-TPFP sites are reduced to the Co^II^ state at close potentials. Subsequent reaction with an O_2_ molecule will generate the peroxo-bridged dinuclear Co^III^ intermediate. The different redox behaviors of **1** and **2** indicated that the Co^III^-TPP unit is likely responsible for the stabilization of the O_2_ adduct and thus for the improved ORR performance of **1**.

In addition to assisting the formation of formally Co^III^–O_2_˙^–^ species, the positively charged Co^III^-TPP unit in the vicinity will also benefit the subsequent 1e reduction of the superoxo unit to generate peroxo, leading to the formation of Co^III^–O_2_^2–^–Co^III^ species. Note that there are two possible pathways for this 1e reduction: the superoxo may be reduced directly, or the Co^III^-TPP is first reduced to its Co^II^ oxidation state and then transfers this electron to the superoxo. After the formation of peroxo, for both **1** and **2**, the peroxo-bridged dinuclear Co^III^ intermediates will be further reduced by two electrons to break the O–O bonds.

Comparing complex **1** with reported dinuclear Co bisporphyrins shows the state-of-the-art ORR performance of **1** (Table 1). The *n* value catalyzed with **1** is 3.90. This value is larger than the *n* values obtained with previous dinuclear Co bisporphyrins. In addition to the increased selectivity, the ORR half-wave overpotential of 510 mV with **1** is also smaller than the half-wave overpotentials obtained with previous dinuclear Co bisporphyrins. These results clearly demonstrate that **1** is superior to other dinuclear Co bisporphyrins in catalyzing the 4e ORR with higher activity and selectivity.

Comparison of **1** with other dinuclear catalysts, including dinuclear Co biscorrole dyads, dinuclear Co porphyrin-corrole dyads, and heterobimetallic Fe/Mn porphyrin-Co corrole dyads, is also made (Table 1). Three conclusions can be obtained from this table. First, heterobimetallic Fe/Mn porphyrin-Co corrole dyads show quite poor selectivity for the 4e ORR, although their half-wave overpotentials are modest among all catalysts. Second, dinuclear Co biscorroles show moderate selectivity for the 4e ORR, indicating the presence of parallel 4e and 2e ORR pathways. Third, dinuclear Co porphyrin-corrole dyads display both high activity and selectivity for the 4e ORR. For dinuclear Co bisporphyrins, only complex **1** can show comparable performance to the dinuclear Co porphyrin-corrole dyads, while other dinuclear Co bisporphyrins all show relatively larger half-wave overpotentials for the ORR. By using electrochemical and spectroscopic methods, Kadish and co-workers established that the catalytically active species of Co corroles for the ORR are generated at relatively more anodic potentials than Co porphyrins with similar substituents.^48^–^50^ As a consequence, dinuclear catalysts, which bear Co corrole moieties, in general will display smaller half-wave overpotentials than dinuclear Co bisporphyrins. Therefore, it is remarkable that the ORR performance of dinuclear Co bisporphyrin **1** (*η*_*E*_1/2__ = 510 mV, *n* = 3.9) is almost identical to that of the dinuclear Co porphyrin-corrole dyad (PCA)Co_2_ (*η*_*E*_1/2__ = 520 mV, *n* = 3.9, Table 1), in terms of both activity and selectivity.

In summary, we report the synthetic procedures and electrocatalytic ORR features of asymmetrical Pacman dinuclear Co porphyrin–porphyrin dyad **1**. Complex **1** is highly efficient and selective to catalyze the 4e ORR in aqueous solutions, and its performance is superior to those of corresponding mononuclear Co porphyrins and also all other symmetrical dinuclear Co bisporphyrins reported in this work and in the literature. The significantly improved performance is due to the asymmetrical structure, in which one Co porphyrin moiety functions as a positively charged Lewis acid to assist the binding and activation of the negatively charged O_2_-adduct unit at its Pacman cleft. This work demonstrates the benefit of asymmetrical dinuclear metal catalysts in electrocatalytic reduction of O_2_, and this catalyst design strategy may be valuable to be explored in other small molecule activation reactions.

## Experimental section

### General materials and methods

Mononuclear Co porphyrins **3** and **4** were synthesized according to reported procedures.^19^,^24^ Synthetic details and characterization of dinuclear Co porphyrins **1** and **2** and also dinuclear Ni porphyrin **5** are described in the ESI.† All reagents were purchased from commercial suppliers and were used as received. Dry solvents, including dimethylformamide, dichloromethane, acetonitrile, and tetrahydrofuran, were purified by passage through activated alumina. Manipulations of air and moisture-sensitive materials were performed under nitrogen gas using standard Schlenk line techniques. All aqueous solutions were prepared freshly using Milli-Q water. NMR measurements were made on a Brüker spectrometer operating at 400 MHz. High-resolution mass spectra were acquired using a Brüker Fourier transform ion cyclotron resonance mass spectrometer APEX IV. UV-vis spectra were recorded using a Cary 50 spectrophotometer. EPR measurements were carried out on a Bruker EMX plus 10/12 CW X-band EPR spectrometer at a microwave frequency of 9.43 GHz using a liquid nitrogen cooling system. The EPR spectra were measured at 90 K with a modulation amplitude of 0.6 mT, a modulation frequency of 100 kHz, and a microwave power of 6.325 mW. Typically, ∼5 mg of sample of complex **1** or **2** was transferred into an EPR tube in a dry box. A toluene solution with a concentration of 1.63 μM for **1** and 1.39 μM for **2** is used for measurements.

### Crystallographic studies

Complete datasets for **2** (CCDC ; 1956352) and **5** (CCDC ; 1956353) were collected. Single crystals suitable for X-ray analysis were each coated with Paratone-N oil, suspended in a small fiber loop, and placed at 150(2) K on a Bruker D8 Venture X-ray diffractometer. Diffraction intensities were measured using graphite monochromated Mo Kα radiation (*λ* = 0.71073 Å). Data collection, indexing, data reduction, and final unit cell refinements were carried out using APEX2;^62^ absorption corrections were applied using the program SADABS.^63^ The structure was solved by direct methods using SHELXS^64^ and refined against *F*^2^ on all data by full-matrix least-squares using SHELXL,^65^ following established refinement strategies. In the X-ray structure of **2** and **5**, all non-hydrogen atoms were refined anisotropically. All hydrogen atoms binding to carbon were included in the model at geometrically calculated positions and refined using a riding model. The isotropic displacement parameters of all hydrogen atoms were fixed to 1.2 times the *U* value of the atoms they are linked to (1.5 times for methyl groups). Details of the data quality and a summary of the residual values of the refinements are listed in ESI Table S1.†

### Electrochemical studies

All electrochemical experiments were carried out using a CH Instruments electrochemical analyzer (model CHI660E) at room temperature. The solution was bubbled with N_2_ gas for at least 30 min before analysis. CVs were recorded in benzonitrile (0.1 M Bu_4_NPF_6_) using a three-compartment cell possessing a 0.07 cm^2^ GC electrode as the working electrode, graphite rod as the auxiliary electrode, and Ag/AgNO_3_ as the reference electrode (BASi, 10 mM AgNO_3_, 0.1 M Bu_4_NPF_6_ in acetonitrile). Ferrocene was added at the end of the measurement as an internal standard. In aqueous solutions, an Ag/AgCl (KCl saturated) electrode was used as the reference electrode. All potentials measured in aqueous solutions are referenced to the reversible hydrogen electrode (RHE). The GC working electrode was polished with α-Al_2_O_3_ of decreasing sizes (1.0 μm to 50 nm) and washed with distilled water and absolute ethanol. For rotating ring-disk electrode measurements, a bipotentiostat (Model CHI 832 Electrochemical Analyzer) and a rotating ring-disk electrode with a rotating GC disk electrode and a platinum ring electrode (ALS RRDE-2) were used. The disk electrode was scanned from 1.20 to 0 V, while the ring potential was held at 1.15 V to detect H_2_O_2_ that was possibly generated during the ORR. The collection efficiency of the ring-disk electrode was evaluated with the [Fe(CN)_6_]^3–/4–^ redox couple and was calculated to be 0.47. Stock dimethylformamide solutions (2 mL) were prepared by mixing multi-walled carbon nanotubes (2 mg), Nafion (20 μL 5%), and Co porphyrin catalysts with the final concentration of 0.1 mM for **1** and **2** and 0.2 mM for **3** and **4**. The mixture solution was sonicated for 30 min, and then well-mixed solutions were applied to the surface of the GC (5 μL) and the GC disk (10 μL) electrodes. Evaporation of the solvents at room temperature produced thin catalyst films.

## Conflicts of interest

The authors declare no competing financial interests.

## Supplementary Material

Supplementary information

Crystal structure data
