# Supplementary material for: Significantly improved electrocatalytic oxygen reduction by an asymmetrical Pacman dinuclear cobalt(ii) porphyrin–porphyrin dyad
Source: Chem Sci. 2019 Nov 4;11(1):87–96. doi: 10.1039/c9sc05041h (PMC7012046; doi:10.1039/c9sc05041h)
Supplement: Supplementary file 1 [file SC-011-C9SC05041H-s001.pdf]

Electronic Supplementary Material (ESI) for Chemical Science.
This journal is © The Royal Society of Chemistry 2019

Electronic Supplementary Information

Significantly improved electrocatalytic oxygen reduction by asymmetrical Pacman dinuclear cobalt(II) porphyrin-porphyrin dyad

Yanju Liu,[1,2] Guojun Zhou,[1] Zongyao Zhang,[2] Haitao Lei,[1] Zhen Yao,[3] Jianfeng Li,[3] Jun Lin,[2] and Rui Cao[1,*]

[1]*Key Laboratory of Applied Surface and Colloid Chemistry, Ministry of Education, School of Chemistry and Chemical Engineering, Shaanxi Normal University, Xi'an 710119, China*

[2]*Department of Chemistry, Renmin University of China, Beijing 100872, China*

[3]*College of Materials Science and Optoelectronic Technology, University of Chinese Academy of Science, Beijing 101408, China*

Correspondence E-mail: ruicao@ruc.edu.cn

Synthesis of dinuclear metal complexes. The synthetic routes of bisporphyrin macrocycles and corresponding metal complexes are depicted in Fig. 2 in the main text. Detailed synthetic conditions and characterizations are described in the following.

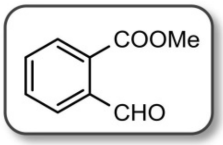

Synthesis of methyl 2-formylbenzoate. *o*-Carboxybenzaldehyde (1.5 g, 9.1 mmol) was dissolved in 200 mL methanol in a 500 mL flask, and 1 mL concentrated sulfuric acid was added with stirring. The mixture was stirred and refluxed for 4 h, and was then added dichloromethane and water for extraction. The dichloromethane layer was separated and was dried with anhydrous Na_2SO_4. Dichloromethane was then removed by vacuo to give methyl 2-formyalbenzoate (1.55 g, yield 95%). ^{1}H NMR (400 MHz, $CDCl_3$): δ = 10.62 (s, 1H), 7.97 (m, 1H), 7.94 (m, 1H), 7.65 (m, 2H), 3.98 (s, 3H) (Fig. S1).

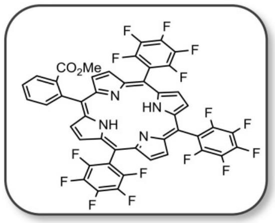

Synthesis of a. To a 500 mL flask containing chloroform (300 mL), were added methyl 2-formylbenzoate (0.41 g, 2.5 mmol), pentafluorobenzaldehyde (1.47 g, 7.5 mmol) and pyrrole (0.67 g, 10 mmol) at room temperature. The solution was purged with N_2 for 30 min, and then $BF_3 \cdot OEt_2$ (0.125 mL, 1 mmol) was added. The mixture was stirred under N_2 for 40 min, and then DDQ (2.04 g, 18 mmol) dissolved in benzene (20 mL) was added. The resulted solution was further stirred for 1 h, and was then dried using a rotary evaporator. The dark residue was dissolved in CH_2Cl_2, and purified by silica-gel column chromatography (hexanes/CH_2Cl_2 = 5:1 v/v) to afford pure **a** as a purple solid (0.47 g yield 20%). ^{1}H NMR (400 MHz, $CDCl_3$): δ = 8.94 (s, 4H), 8.83 (s, 4H), 8.49 (d, 1H),

8.18 (d, 1H), 7.87-7.97 (m, 2H), 2.89 (s, 3H), −2.67 (s, 2H) (Fig. S2). HRMS of $[M+H]^+$: calcd. for $C_{46}H_{18}F_{15}N_4O_2$, 943.1185; found, 943.1179 (Fig. S3).

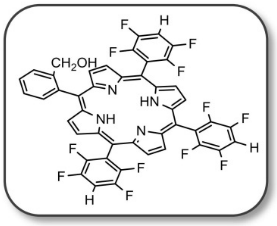

Synthesis of b. To a dry THF (50 mL) solution of **a** (0.8 g, 0.82 mmol) in an ice bath, $LiAlH_4$ (150 mg, 3.5 mmol) was added slowly. The mixture was stirred for 5 h at temperature. Methanol (20 mL) and water (15 mL) were then added to quench the reaction. The resulted mixture was extracted with dichloromethane, washed with water (50 mL × 3), and dried with anhydrous Na_2SO_4. The solution was concentrated to dryness using a rotary evaporator to afford **b** as a solid (0.64 g, yield 87%). ^1H NMR (400 MHz, $CDCl_3$): δ = 8.92 (s, 4H), 8.82 (s, 4H), 8.08 (d, 1H), 7.98 (d, 1H), 7.88 (t, 1H), 7.70 (t, 1H), 7.65-7.57 (m, 3H), 4.35 (s, 2H), −2.78 (s, 2H) (Fig. S4). HRMS of $[M+H]^+$: calcd. for $C_{45}H_{21}F_{12}N_4O$, 861.1518; found, 861.1532 (Fig. S5).

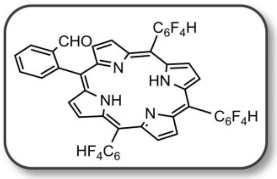

Synthesis of c. Dry dichloromethane (2.5 mL) was added to a dried Schlenk flask (100 mL) containing oxalyl chloride (47 uL, 0.55 mmol) under N_2 and −78 °C. DMSO (78 uL, 1.1 mmol) was added slowly, and the mixture was stirred for 15 min at −78 °C. Complex **b** (460 mg, 0.5 mmol) dissolved in dry dichloromethane (30 mL) was then added slowly, and the resulted solution was stirred for 5 min. Next, dry triethylamine (605 uL, 4.35 mmol) was added, and was stirred for another 10 min at −78 °C. The solution was then kept stirring at room temperature for overnight. The reaction was quenched with CH_3OH and H_2O, and was extracted with dichloromethane, washed with water (50 mL × 3), and was dried with

anhydrous Na_2SO_4. The solution was concentrated to dryness using a rotary evaporator to afford **c** as a solid (0.422 g, yield 92%). ^1H NMR (400 MHz, $CDCl_3$): δ = 9.50 (s, 1H), 8.92 (s, 4H), 8.76-8.85 (s, 4H), 8.44 (d, 1H), 8.23 (d, 1H), 8.03-7.93 (m, 2H), 7.68-7.57 (m, 3H), −2.73 (s, 2H) (Fig. S6). HRMS of $[M+H]^+$: calcd. for $C_{45}H_{19}F_{12}N_4O$, 859.1362; found, 859.1355 (Fig. S7).

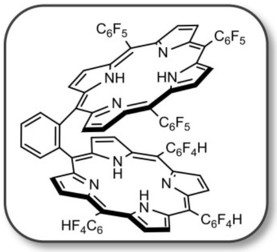

Synthesis of 5'. To a 500 mL flask containing chloroform (300 mL), were added **c** (0.422 g, 0.5 mmol), benzaldehyde (582 uL, 5.5 mmol), and pyrrole (416 uL, 6.0 mmol) at room temperature. After purging with argon for 30 min, $BF_3{\cdot}OEt_2$ was added. The mixture was stirred under argon for 40 min, and then DDQ dissolved in benzene (20 mL) was added, and the solution was stirred for 1 h. The reaction solution was dried using a rotary evaporator. The resulted dark residue was dissolved in dichloromethane, and purified by silica-gel column chromatography (hexanes/dichloromethane = 5:1 v/v) to afford pure **5'** as a purple solid (96.5 mg, yield 12%). ^1H NMR (400 MHz, $CDCl_3$): δ = 9.37-9.32 (m, 4H), 8.89-8.82 (m, 2H), 8.48-8.34 (m, 14H), 7.63-7.52 (m, 2H), 7.45-7.35 (m, 1H), −4.03 (s, 4H) (Fig. S8). HRMS of $[M+H]^+$: calcd. for $C_{82}H_{28}F_{27}N_8$, 1637.2000; found, 1637.1995 (Fig. S9).

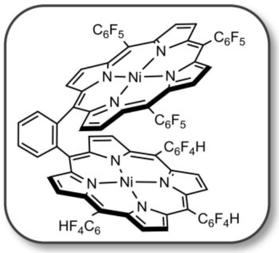

Synthesis of 5. The mixture of **5'** (96 mg, 60 umol) in 50 mL DMF was treated with $Ni(OAc)_2{\cdot}4H_2O$ (75 mg, 0.3 mmol). The reaction mixture was kept stirring and heating at 115 °C under argon for 4 h. The solvent was then removed under reduced

pressure, and the resulted dark solid was dissolved in dichloromethane and washed with distilled water. The organic phase was concentrated to dryness and was subjected to silica chromatography (hexane:dichloromethane = 5:1) to afford red solid of **5** (92 mg, yield 87%). HRMS of $[M+Na]^+$: calcd. for $C_{82}H_{23}F_{27}N_8NaNi_2$, 1771.0214; found, 1771.0225 (Fig. S10).

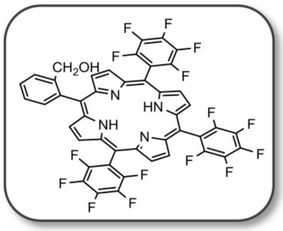

Synthesis of d. To a dried Schlenk flask (100mL) containing dry toluene solution (60 mL) of **a** (550 mg, 0.58 mmol) under N_2 and −78 °C, DIBAL-H (2 mL, 1.2 mmol) was added. The solution was stirred for 5 h under N_2 and −78 °C, and was quenched by 20 mL CH_3OH and 30 mL H_2O. The mixture was added 30 mL saturated sodium potassium tartrate solution and stirred for overnight. Next, the solution was extracted by dichloromethane, and was dried with anhydrous Na_2SO_4. The resulted solution was dried using a rotary evaporator to afford a purple solid compound **d** (519 mg, yield 95%). ^1H NMR (400 MHz, $CDCl_3$): δ = 8.91 (s, 4H), 8.82 (s, 4H), 8.08 (d, 1H), 7.98 (d, 1H), 7.88 (t, 1H), 7.70 (t, 1H), 4.35 (s, 2H), −2.80 (s, 2H) (Fig. S11). HRMS of $[M+Na]^+$: calcd. for $C_{45}H_{17}F_{15}N_4NaO$, 937.1055; found, 937.1095 (Fig. S12).

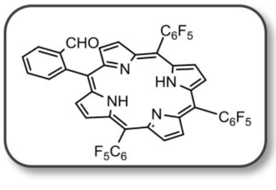

Synthesis of e. Dry dichloromethane (2.5 mL) was added to a dried Schlenk flask (100mL) containing oxalyl chloride (47 uL, 0.55 mmol) under N_2 and −78 °C. DMSO (78 uL, 1.1 mmol) was added slowly, and the mixture was stirred for 15 min under N_2 and −78 °C. Next, complex **d** (460 mg, 0.5 mmol) dissolved in dry dichloromethane (30 mL) was added to

the mixture slowly and was stirred for 5 min. Dry triethylamine (605 uL, 4.35 mmol) was added to the above solution and was stirred for 10 min under N_2 and −78 °C. The solution was then kept stirring at room temperature for overnight. The reaction was quenched with CH_3OH and H_2O. The mixture was extracted with dichloromethane, washed with water (50 mL × 3), and was dried with anhydrous Na_2SO_4. The solution was concentrated to dryness using a rotary evaporator to afford **e** as a solid (416 mg, yield 93%). ^{1}H NMR (400 MHz, $CDCl_3$): δ = 9.50 (s, 1H), 8.92 (s, 4H), 8.86-8.76 (s, 4H), 8.44 (d, 1H), 8.23 (d, 1H), 8.05-7.94 (m, 2H), −2.77 (s, 2H) (Fig. S13). HRMS of $[M+H]^+$: calcd. for $C_{45}H_{16}F_{15}N_4O$, 913.1079; found, 913.1085 (Fig. S14).

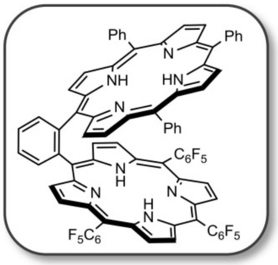

Synthesis of 1'. To a 500 mL flask containing chloroform (300 mL), were added **e** (0.416 g, 0.5 mmol), benzaldehyde (582 uL, 5.5 mmol), and pyrrole (416 uL, 6.0 mmol) at room temperature. After purging with argon for 20 min, $BF_3{\cdot}OEt_2$ was added. The mixture was stirred under argon for 40 min. Then DDQ dissolved in benzene (20 mL) was added and the solution was further stirred for 1 h. The solution was dried using a rotary evaporator, and the resulted dark residue was dissolved in dichloromethane, and purified by silica-gel column chromatography (hexanes/dichloromethane = 5:1 v/v) to afford pure **1'** as a purple solid (85 mg, yield 13%). ^{1}H NMR (400 MHz, $CDCl_3$): δ = 9.49 (d, 2H), 9.29 (d, 2H), 8.86 (m, 2H), 8.56 (d, 2H), 8.52 (d, 2H), 8.47 (s, 4H), 8.42 (s, 4H), 8.34 (m, 2H), 8.04 (s, 1H), 7.81 (m, 8H), 7.64-7.58 (m, 4H), 7.52-7.40 (m, 2H), −3.91 (m, 4H) (Fig. S15). HRMS of $[M+H]^+$: calcd. for $C_{82}H_{40}F_{15}N_8$, 1421.3131; found, 1421.3137 (Fig. S16).

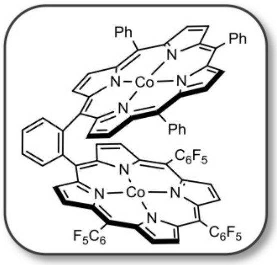

Synthesis of 1. Complex **1'** (85 mg, 60 umol) was dissolved in 50 mL DMF, and was then treated with $CoCl_2$ (40 mg, 0.3 mmol). The reaction solution was kept stirring and heating at 115 °C under argon for 4 h. Next, the solvent was removed under reduced pressure, and the resulted dark solid was dissolved in dichloromethane and washed with water. The organic phase was concentrated to dryness and subjected to silica chromatography (hexane:dichloromethane = 5:1) to afford red solid of **1** (80 mg, yield 87%). HRMS of $[M]^+$ and $[M+H]^+$ are overlapped: calcd. for $C_{82}H_{35}Co_2F_{15}N_8$, 1534.1404 and $C_{82}H_{36}Co_2F_{15}N_8$, 1535.1437; found, 1534.1404 and 1535.1461 (Fig. S17).

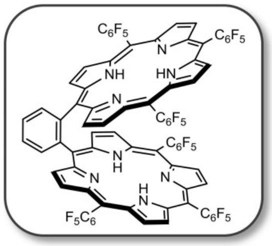

Synthesis of 2'. The synthetic conditions of **2'** are the same to that of **1'**, except the use of pentafluorobenzaldehyde (yield 16%). ^{1}H NMR (400 MHz, $CDCl_3$): δ = 9.33 (t, 4H), 8.87-8.82 (m, 2H), 8.46-8.82 (m, 14H), −4.04 (s, 4H) (Fig. S18). HRMS of $[M+H]^+$: calcd. for $C_{82}H_{25}F_{30}N_8$, 1691.1718; found, 1691.1699 (Fig. S19).

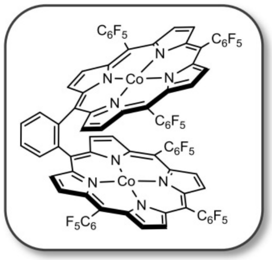

Synthesis of 2. The synthetic conditions of **2** are the same to that of **1** mentioned above, except the use of **2'** as the porphyrin ligand (yield 89%). HRMS of $[M]^+$ and $[M+H]^+$ are overlapped: calcd. for $C_{82}H_{20}Co_2F_{30}N_8$, 1803.9990 and $C_{82}H_{21}Co_2F_{30}N_8$, 1805.0023; found, 1803.9995 and 1805.0038 (Fig. S20).

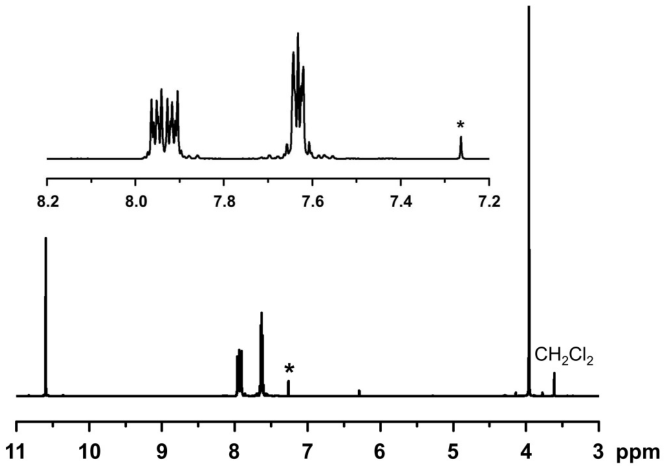

Fig. S1 ^{1}H NMR spectrum of methyl 2-formylbenzoate in $CDCl_3$. The solvent residue peak of $CDCl_3$ is labeled (*).

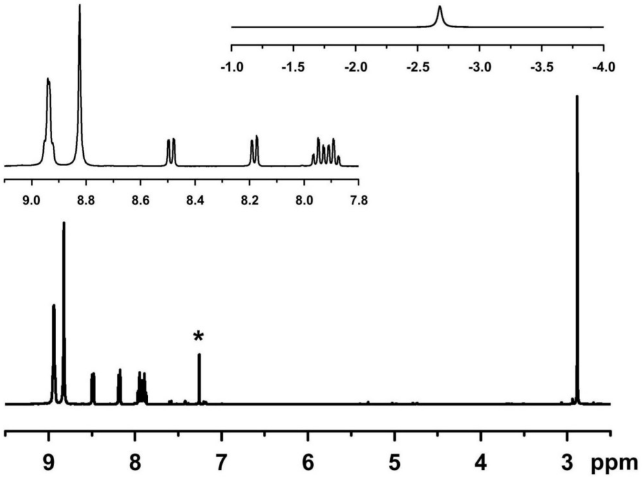

Fig. S2 ^{1}H NMR spectrum of **a** in $CDCl_3$. The solvent residue peak of $CDCl_3$ is labeled (*).

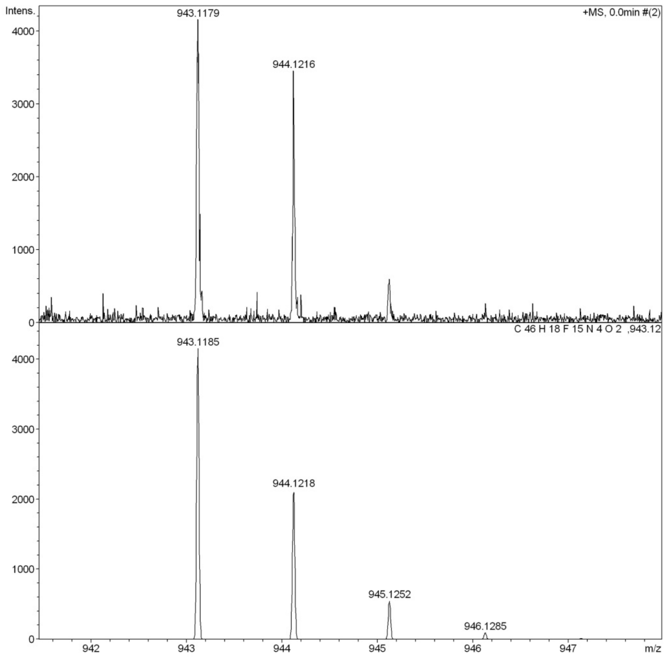

Fig. S3 HRMS of **a**. The ion at a mass-to-charge ratio of 943.1179 matches the calculated value of 943.1185 for the monocation of $[C_{46}H_{18}F_{15}N_4O_2]^+$ with identical isotopic distribution pattern.

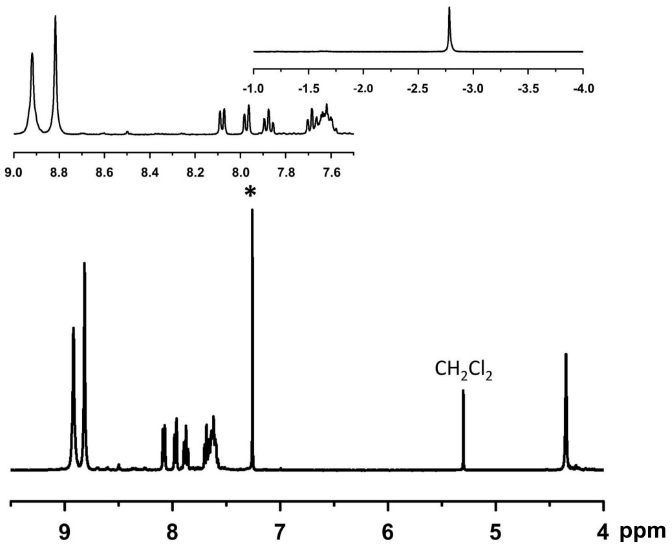

Fig. S4 ^{1}H NMR spectrum of **b** in $CDCl_3$. The solvent residue peak of $CDCl_3$ is labeled (*).

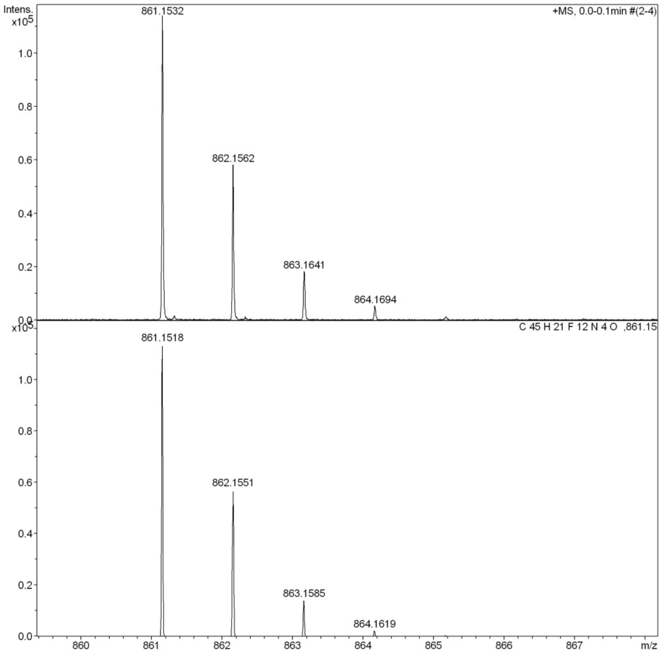

Fig. S5 HRMS of **b**. The ion at a mass-to-charge ratio of 861.1532 matches the calculated value of 861.1518 for the monocation of $[C_{45}H_{21}F_{12}N_4O]^+$ with identical isotopic distribution pattern.

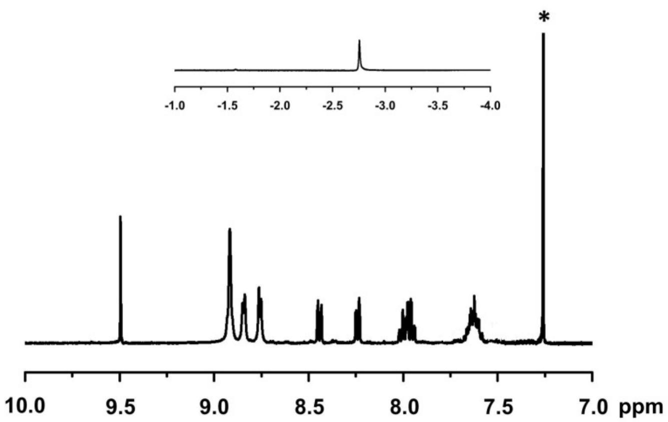

Fig. S6 ^{1}H NMR spectrum of **c** in $CDCl_3$. The solvent residue peak of $CDCl_3$ is labeled (*).

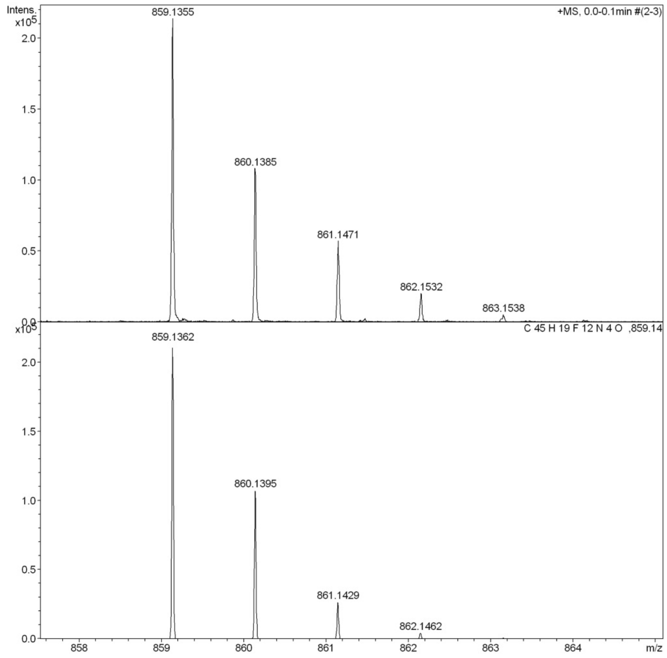

Fig. S7 HRMS of **c**. The ion at a mass-to-charge ratio of 859.1355 matches the calculated value of 859.1362 for the monocation of $[C_{45}H_{19}F_{12}N_4O]^+$ with identical isotopic distribution pattern.

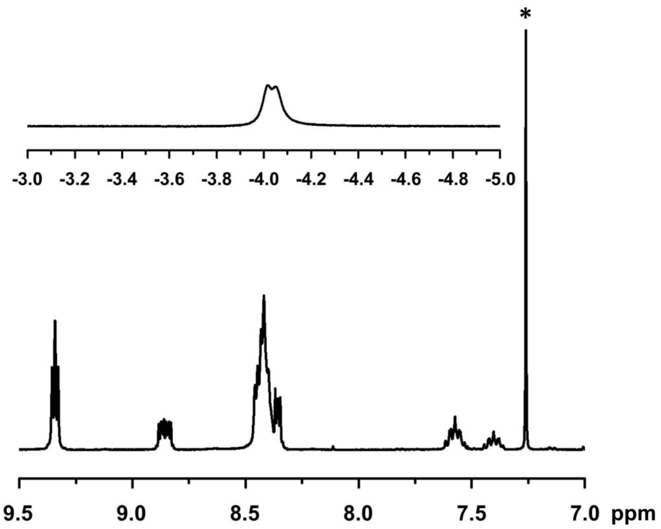

Fig. S8 ^{1}H NMR spectrum of **5'** in $CDCl_3$. The solvent residue peak of $CDCl_3$ is labeled (*).

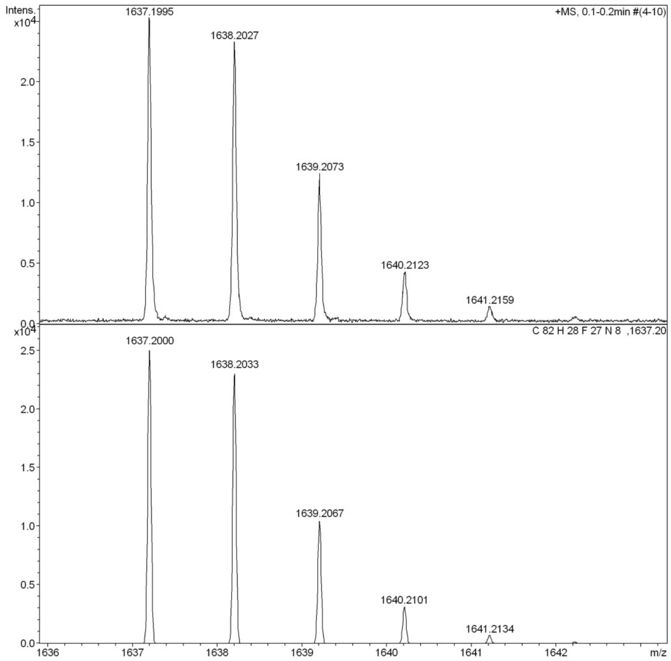

Fig. S9 HRMS of **5’**. The ion at a mass-to-charge ratio of 1637.1995 matches the calculated value of 1637.2000 for the monocation of $[C_{82}H_{28}F_{27}N_8]^+$ with identical isotopic distribution pattern.

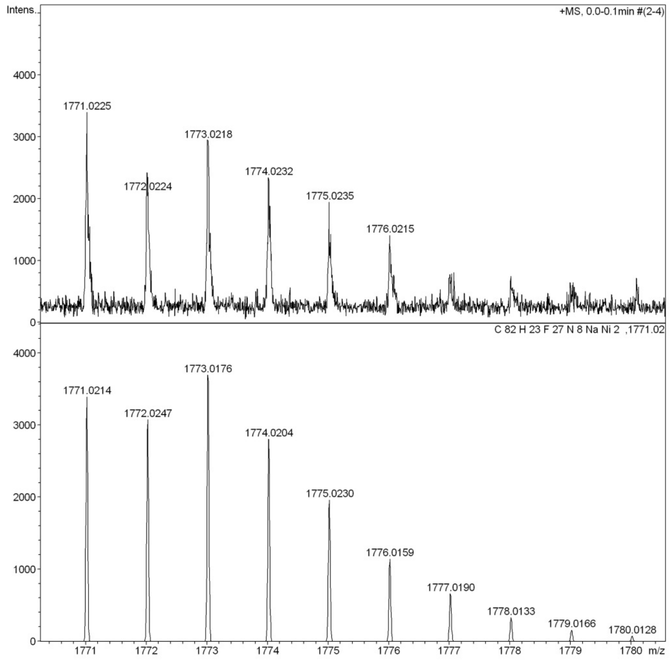

Fig. S10 HRMS of **5**. The ion at a mass-to-charge ratio of 1771.0225 matches the calculated value of 1771.0214 for the monocation of $[C_{82}H_{23}F_{27}N_8NaNi_2]^+$ with identical isotopic distribution pattern.

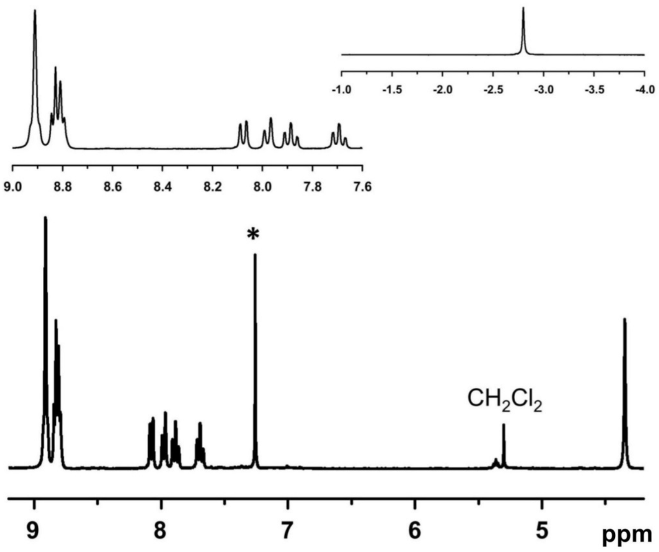

Fig. S11 ^{1}H NMR spectrum of **d** in $CDCl_3$. The solvent residue peak of $CDCl_3$ is labeled (*).

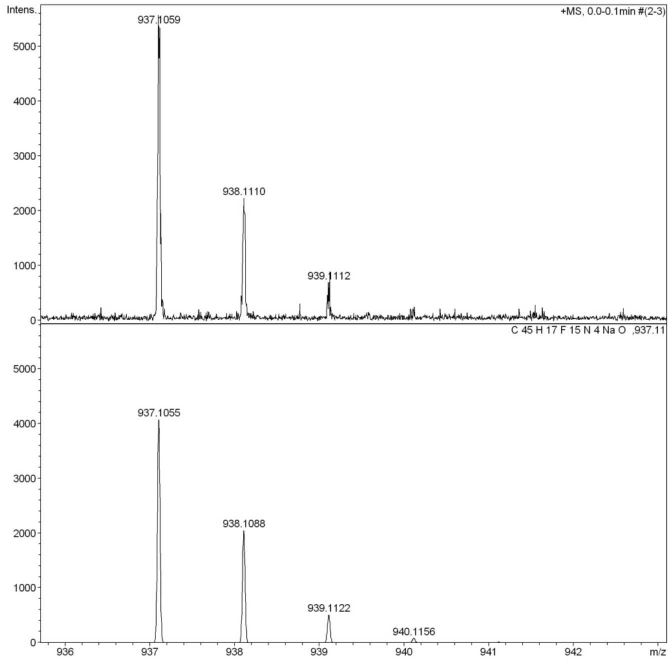

Fig. S12 HRMS of **d**. The ion at a mass-to-charge ratio of 937.1059 matches the calculated value of 937.1055 for the monocation of $[C_{45}H_{17}F_{15}N_4NaO]^+$ with identical isotopic distribution pattern.

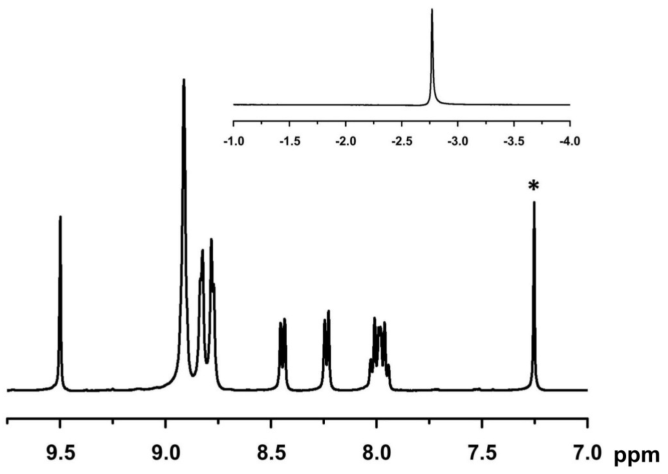

Fig. S13 ^{1}H NMR spectrum of **e** in $CDCl_3$. The solvent residue peak of $CDCl_3$ is labeled (*).

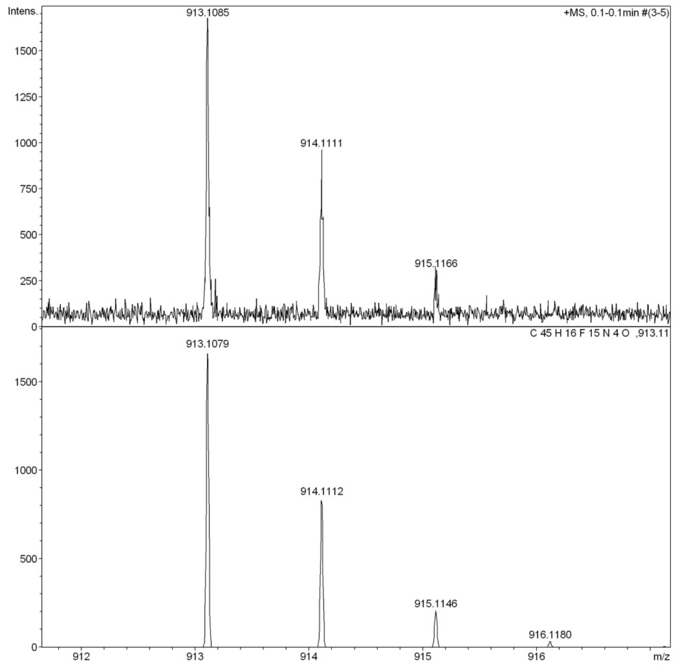

Fig. S14 HRMS of **e**. The ion at a mass-to-charge ratio of 913.1085 matches the calculated value of 913.1079 for the monocation of $[C_{45}H_{16}F_{15}N_4O]^+$ with identical isotopic distribution pattern.

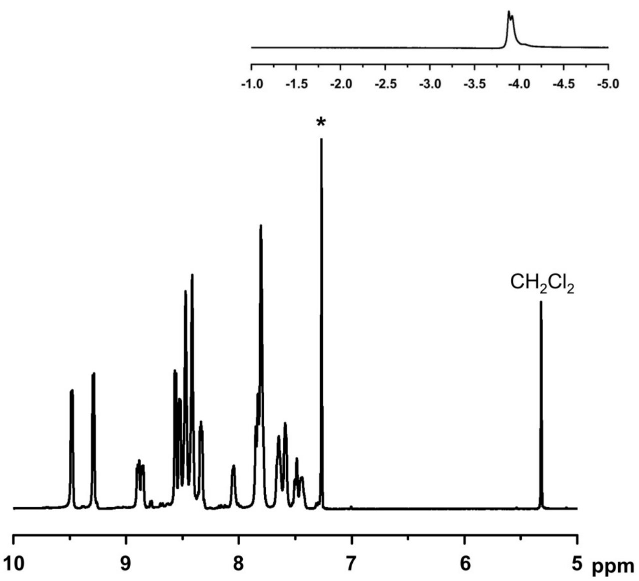

Fig. S15 ^{1}H NMR spectrum of **1'** in $CDCl_3$. The solvent residue peak of $CDCl_3$ is labeled (*).

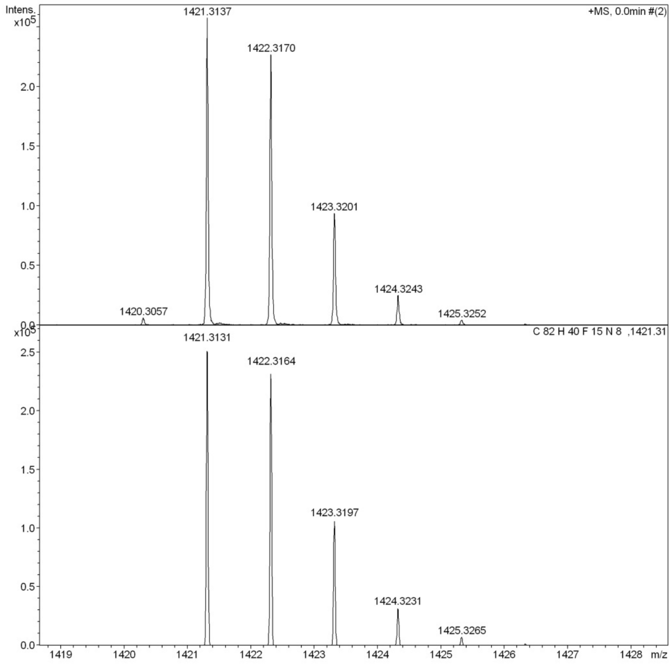

Fig. S16 HRMS of **1'**. The ion at a mass-to-charge ratio of 1421.3137 matches the calculated value of 1421.3131 for the monocation of $[C_{82}H_{40}F_{15}N_8]^+$ with identical isotopic distribution pattern.

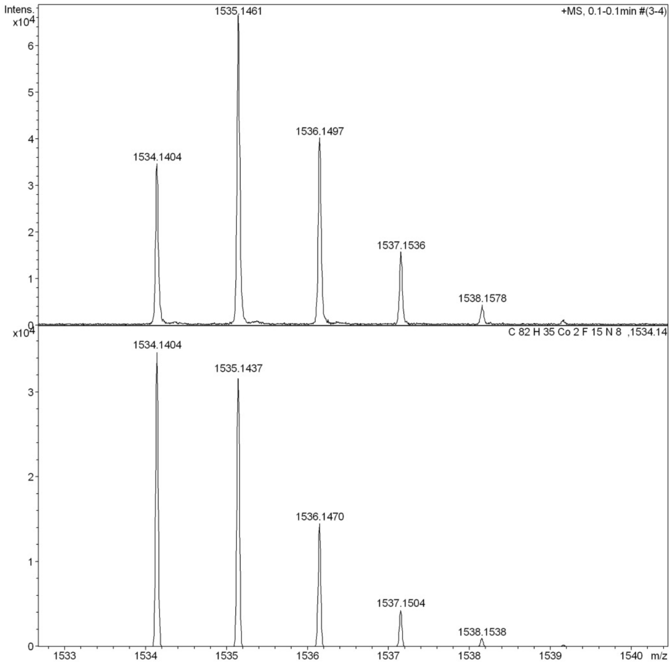

Fig. S17 HRMS of **1**. The ions at a mass-to-charge ratio of 1534.1404 and 1535.1461 matches the calculated value of 1534.1404 and 1535.1437 for the monocation of $[C_{82}H_{35}Co_2F_{15}N_8]^+$ and $[C_{82}H_{36}Co_2F_{15}N_8]^+$ with identical overlapped sum of isotopic distribution patterns.

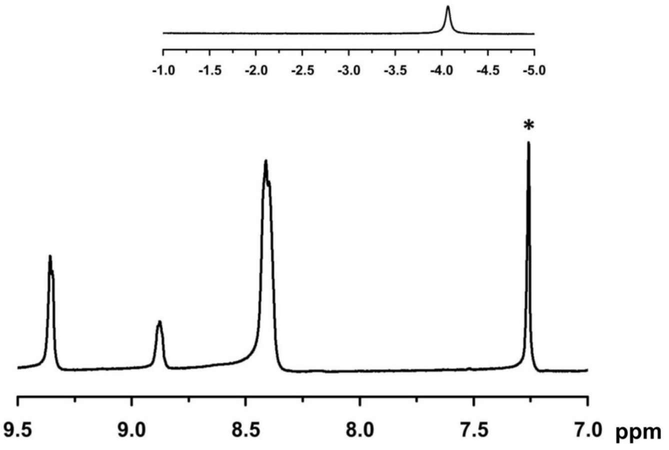

Fig. S18 ^{1}H NMR spectrum of **2'** in $CDCl_3$. The solvent residue peak of $CDCl_3$ is labeled (*).

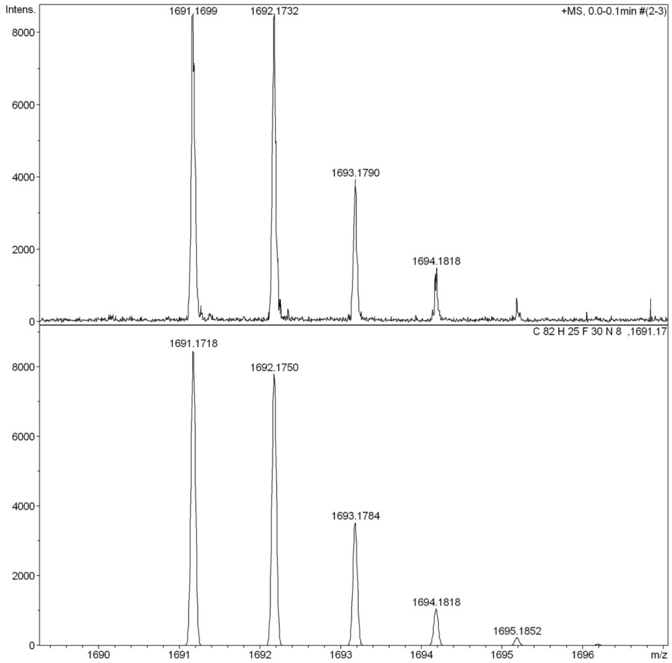

Fig. S19 HRMS of **2'**. The ion at a mass-to-charge ratio of 1691.1699 matches the calculated value of 1691.1718 for the monocation of $[C_{82}H_{25}F_{30}N_8]^+$ with identical isotopic distribution pattern.

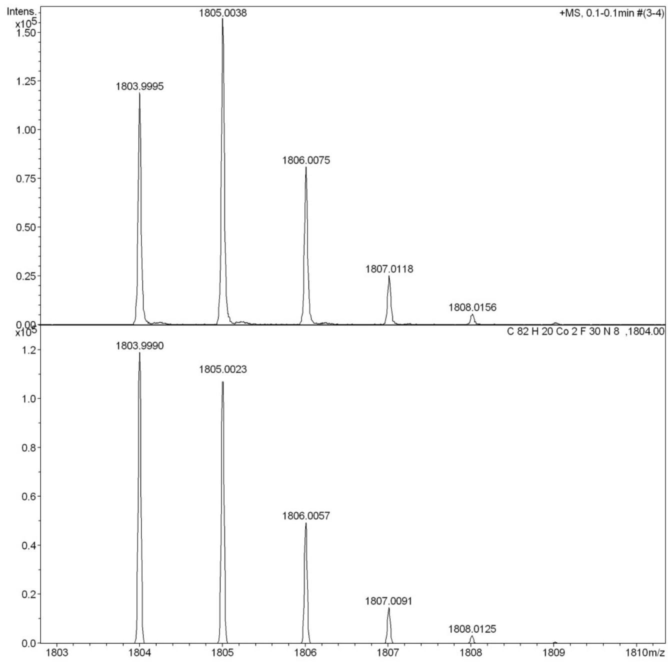

Fig. S20 HRMS of **2**. The ions at a mass-to-charge ratio of 1803.9995 and 1805.0038 matches the calculated value of 1803.9990 and 1805.0023 for the monocation of $[C_{82}H_{20}Co_2F_{30}N_8]^+$ and $[C_{82}H_{21}Co_2F_{30}N_8]^+$ with identical overlapped sum of isotopic distribution patterns.

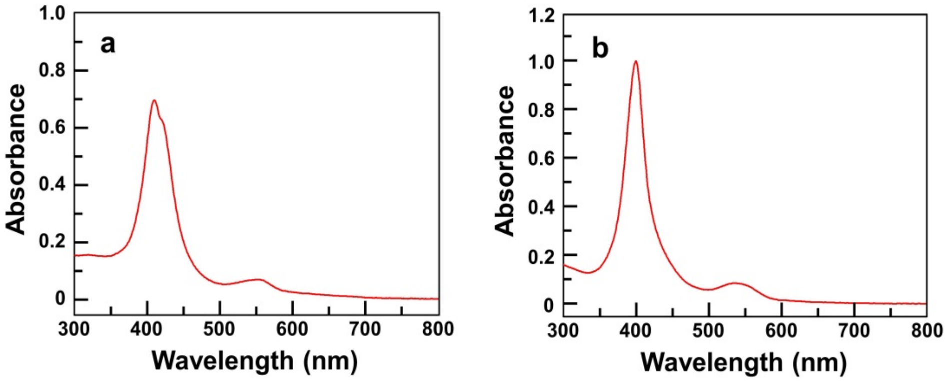

Fig. S21 UV-vis absorption spectra of **1** (a) and **2** (b) in DMF.

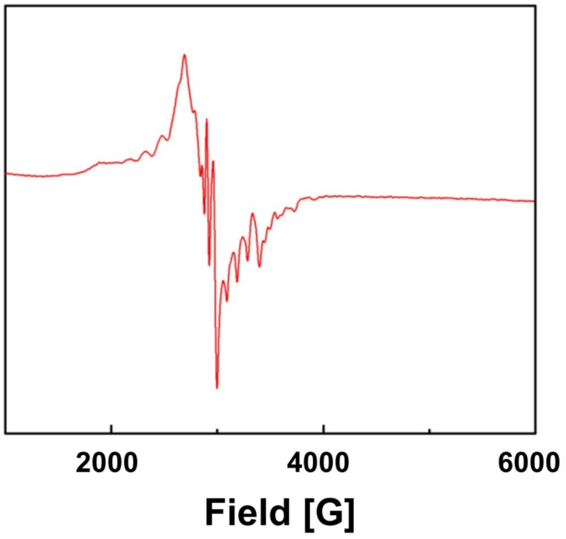

Fig. S22 EPR spectrum of **1** in toluene at 90 K.

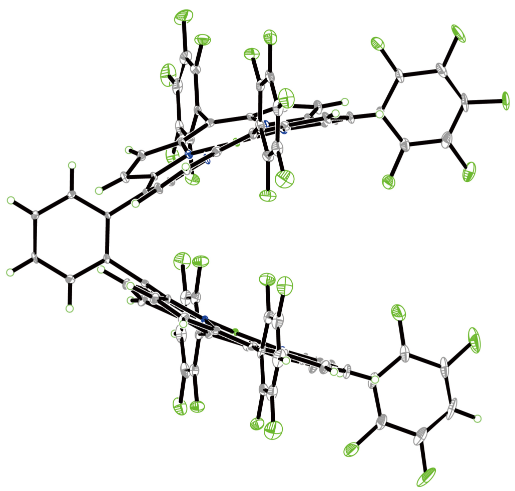

Fig. S23 Thermal ellipsoid plot of the X-ray structure of **5** (30% probability). In the bottom porphyrin macrocycle, the F atom at the *para*-position of three pentafluorophenyl units is replaced by an H atom.

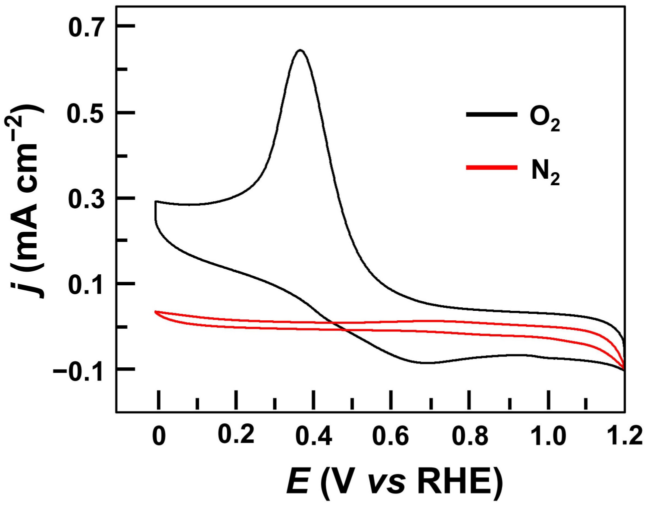

Fig. S24 CVs of **3** in 0.5 M H_2SO_4 under N_2 (red) and O_2 (black). Conditions: GC working electrode, scan rate 100 mV s^{-1}, 20 °C.

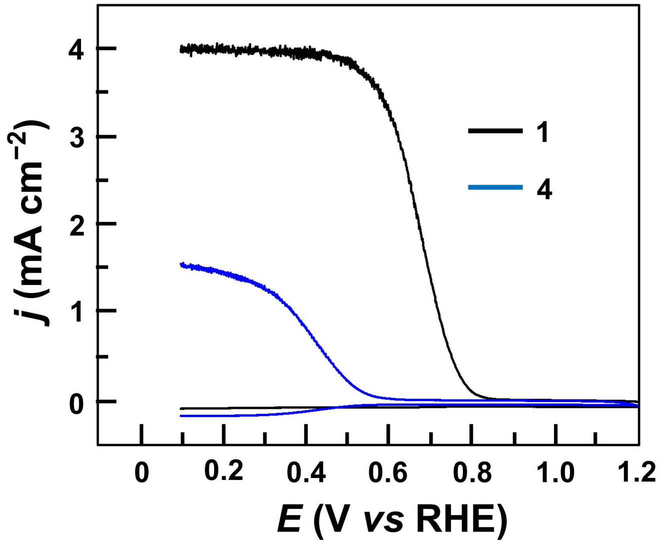

Fig. S25 RRDE measurements for ORR at the GC disk electrode coated with **1** (black) and **4** (blue) in an O_2-saturated 0.5 M H_2SO_4 solution at 2500 rpm. The ring electrode was polarized at 1.0 V.

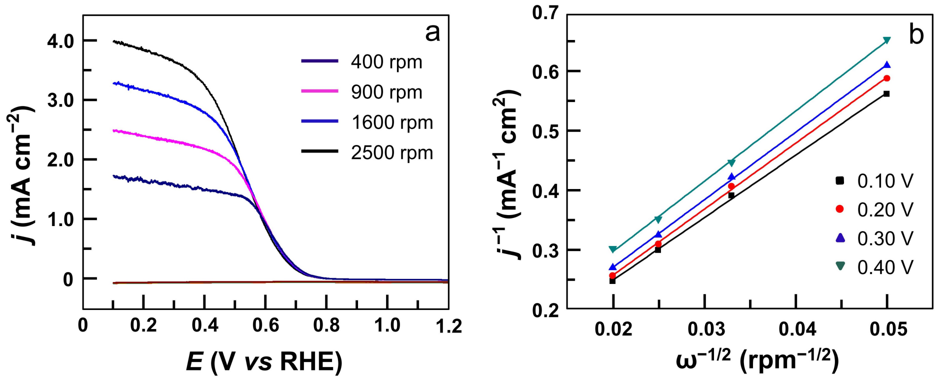

Fig. S26 (a) RRDE measurements for ORR with **2** in O_2-saturated 0.5 M H_2SO_4 solution at various rotation rates. (d) K-L plots for ORR with **2**.

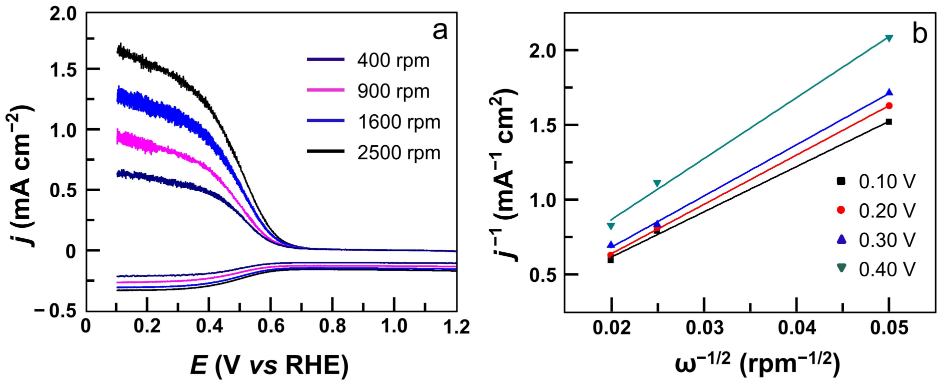

Fig. S27 (a) RRDE measurements for ORR with **3** in O_2-saturated 0.5 M H_2SO_4 solution at various rotation rates. (d) K-L plots for ORR with **3**.

Table S1. Crystal data and structure refinement parameters for the X-ray structures of **2** and **5**.

complex	**2**	**5**
molecular formula	$C_{89}H_{28}Co_2F_{30}N_8$	$C_{93}H_{48}Cl_3F_{27}N_8Ni_2$
formula wt. (g mol^{-1})	1897.05	2014.16
temperature (K)	173(2)	153(2)
radiation (λ, Å)	0.71073	0.71073
crystal system	Triclinic	Triclinic
space group	$P\bar{1}$ (#2)	$P\bar{1}$ (#2)
a (Å)	14.422(2)	14.9706(19)
b (Å)	15.261(3)	15.430(2)
c (Å)	20.632(4)	19.502(2)
α (°)	67.7449(10)	76.905(4)
β (°)	84.949(2)	68.784(4)
γ (°)	74.9246(10)	84.109(5)
Volume (Å^3)	4057.6(12)	4089.3(9)
Z	2	2
ρ_{calcd} (g cm^{-3})	1.553	1.636
μ (mm^{-1})	0.530	0.676
F(000)	1884	2024
crystal size (mm^3)	0.20 × 0.20 × 0.18	0.30 × 0.30 × 0.10
Theta range	1.066 to 25.500°	2.147 to 26.416°
reflections collected	45101	69320
indep. reflections	14661 [R(int) = 0.0967]	16774 [R(int) = 0.1433]
Completeness	97.1%	99.9%
goodness-of-fit on F^2	1.065	1.049
final R indices [R > 2σ (I)]	$R_1{}^a$ = 0.0496<br>$wR_2{}^b$ = 0.1103	$R_1{}^a$ = 0.0791<br>$wR_2{}^b$ = 0.1484
R indices (all data)	$R_1{}^a$ = 0.0572<br>$wR_2{}^b$ = 0.1125	$R_1{}^a$ = 0.1297<br>$wR_2{}^b$ = 0.1705
largest diff. peak and hole (e Å^{-3})	0.666 and −0.565	1.263 and −0.776

$^a R_1 = \Sigma||F_o| - |F_c|| / |F_o|$, $^b wR_2 = \{\Sigma[w(F_o{}^2 - F_c{}^2)^2] / \Sigma[w(F_o{}^2)^2]\}^{0.5}$
